# Supplementary material for: Heat shock-induced chaperoning by Hsp70 is enabled in-cell
Source: PLoS One. 2019 Sep 26;14(9):e0222990. doi: 10.1371/journal.pone.0222990 (PMC6762143; doi:10.1371/journal.pone.0222990)
Supplement: S1 File — Contains supplementary results, supplementary methods, supplementary A through J Figures, and supplementary A through D Tables, highlighting control experiments, protein sequences, and thermodynamic protein data. (PDF) [file pone.0222990.s001.pdf]

Supplementary Information

Heat shock-induced chaperoning by Hsp70 is enabled in-cell

Drishti Guin[1], Hannah Gelman[2,#a], Yuhan Wang[3] and Martin Gruebele[1,2,3,*]

[1]Department of Chemistry, University of Illinois Urbana-Champaign, Urbana, Illinois, United States of America

[2]Department of Physics, University of Illinois Urbana-Champaign, Urbana, Illinois, United States of America

[3]Center for Biophysics and Quantitative Biology, University of Illinois Urbana-Champaign, Urbana, Illinois, United States of America

[#a]Current Address: U.S. Department of Veterans Affairs, Seattle, Washington, United States of America

*Corresponding author

Email: mgruebel@illinois.edu

Supplementary results

PGK-FRET aggregation on thermal unfolding

PGK unfolding was monitored by heating a 5 μM sample of PGK-FRET in a thermally controlled fluorometer. PGK-FRET is labeled at the N-terminus with an mEGFP fluorophore and at the C-terminus with an mCherry fluorophore. The FRET efficiency curve was fit to a three-state model similar to what is shown in equation 5 in the main text except with the three states being folded, unfolded and aggregated (Fig A). The fit yielded an unfolding T_m of 45 °C and an aggregated T_a of 52 °C where PGK-FRET self-aggregates on unfolding.

mHsp70 and mHsp70K71M ATPase activity

The absorbance spectrum of PercevalHR shifts according to the nucleotide present (Fig B, panel A). With 2 mM ATP the absorbance peak at 500 nm increased while with 2 mM ADP the absorbance peak at 420 nm increased. The ATPase activity was measured by monitoring the emission of PercevalHR at 520 for 500 nm excitation (Fig B, panel B). As the ATP is hydrolyzed the absorbance at 500 nm decreases and hence the emission at 520 nm for the 500 nm excitation also decreases. The curves were fit using 1st order reaction kinetics and yielded an ATP hydrolysis rate of 6.31 X 10^{-1} sec^{-1} for mHsp70 and 3.69 X 10^{-2} sec^{-1} mHsp70K71M.

Supplementary methods

In vitro circular dichroism and fluorometer melts

Prior to all measurements the glycerol from the frozen stocks were removed by spin filtration buffer exchange. Tryptophan fluorescence measurements and *in vitro* FRET binding experiments were conducted on an FP8300 spectrofluorometer equipped with Peltier temperature control

(JASCO). For *in vitro* PGK characterization, tryptophan was excited at 295 nm, and emission spectra were collected from 290 to 450 nm. Samples were measured in 300 µL cuvettes at 5 µM concentrations, unless otherwise noted.

Circular dichroism (CD) was measured using a J-715 spectropolarimeter with Peltier temperature control (JASCO). Unless otherwise noted, all spectra were recorded from 250 to 200 nm at a scan rate of 50 nm/min at 1 nm resolution and averaging five accumulations. Thermal melts were done using a 2 mm path length cuvette. Unless otherwise noted, protein concentration in circular dichroism experiments was 5 µM. Protein unfolding was monitored by measuring the change in mean residue ellipticity at 222 nm.

All *in vitro* fluorometer melts were conducted in K1 buffer with 2 mM ATP and 10 mM DTT. For measurements with 1:5 mHsp70:ePGK, 2.5 µM ePGK was mixed with 0.5 µM mHsp70. Control measurements were conducted with either 5:1 mHsp70:mEGFP (5 µM mHsp70 and 1 µM mEGFP) or 5:1 mCherry:ePGKs (5 µM mCherry and 1 µM ePGKs). For crowding Ficoll70 was added to a final concentration of 300 mg/mL along with 1 µM Hsp40. Temperature melts with Ficoll showed signs of aggregation at higher temperatures.

In vitro FRET-PGK1 unfolding with denaturant guanidinium hydrochloride (GuHCl) was measured by conducting isothermal titrations at 20 °C of 5 µM FRET-PGK1 with GuHCl between 0-0.8 M. mEGFP was excited at 485 nm and emission spectra were collected from 480 to 700 nm in 300 μL cuvettes.

Measurement of ATPase assay with PercevalHR

Prior to all measurements the glycerol from the frozen stocks were removed by spin filtration buffer exchange. ATPase assays were conducted with the ATP sensor protein PercevalHR[1]. The plasmid for bacterial expression, pRsetB-PercevalHR (Addgene plasmid # 49081), was obtained from Addgene and was a gift from Gary Yellen. The protein was expressed as described in the main text and dialyzed in 5 mM MOPS, 300 mM NaCl, pH 7.3. Absorbance measurements were made in K1 buffer with 2 mM total nucleotide (2 mM ATP, 2 mM ADP or 1 mM each of ATP and ADP) and 5 μM PercevalHR. ATPase assays were conducted also in K1 buffer with 5 μM mHsp70 or mHsp70K71M, 2.5 μM Hsp40, 10 μM PercevalHR and 1 mM ATP. The PercevalHR emission intensity was monitored at 520 nm for 500 nm excitation for 2 hours at 15 second intervals at 37 °C.

Data analysis

Data analysis was performed with MATLAB (Mathworks). Midpoint denaturant concentration (C_m) was calculated using a two-state sigmoidal fit to the experimental isothermal titration data. Native and denatured state baselines were assumed to be linear with respect to the perturbing variable C (concentration) so that the signal S can be estimated by:

$$1)\ S_i = m_i(C - C_m) + b_i\,,$$

where i is either the native state (N) or the denatured state (D), m is the slope and b is the intercept. C_m is the corresponding midpoint of the curve with the concentration C. The total signal $S(T)$ can then be estimated as a sigmoidal function with respect to concentration, C:

$$2)\quad S(C) = S_N(C)f_N(C) + S_D(C)f_D(C)\,,$$

where, f_N and f_D are the populations of the N and D states, respectively.

$$3)\quad K_{eq} = e^{-\frac{\Delta G_{N \to D}}{RT}},$$

$$4)\quad \Delta G_{N \to D} \approx \delta g_1(\, C - C_m),$$

$$5)\quad f_N = \frac{K_{eq}}{1 + K_{eq}},$$

$$6)\quad and f_D = \frac{1}{1 + K_{eq}}$$

Fits to equation 2 yield the midpoint concentration (C_m).

The kinetic data for mHsp70 ATPase activity was fit using the equation:

$$\frac{(1+a)exp^{-kt}}{2} + \frac{1-a}{2}$$

where, k is the reaction rate. The mHsp70 fits yielded a value of -0.6623 for a which was fixed at this value for fitting the kinetic data for mHsp70K71M. The data for mHsp70K71M was then fit to the equation:

$$\frac{(1+0.6623)exp^{-kt}}{2} + \frac{1-0.6623}{2}$$

References

1. Tantama, M., Martínez-François, J. R., Mongeon, R. & Yellen, G. Imaging energy status in live cells with a fluorescent biosensor of the intracellular ATP-to-ADP ratio. *Nat. Commun.* **4,** (2013).

Supplementary Figures

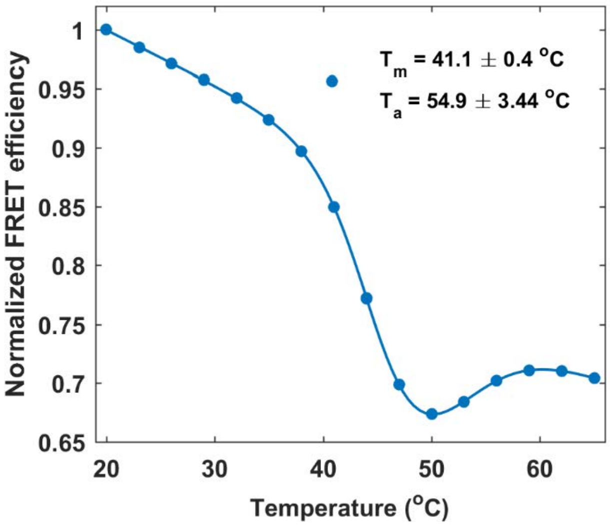

Figure A: The thermal unfolding of PGK monitored by FRET efficiency. Filled markers show experimental data and solid line shows fit to the experimental data. PGK unfolds with a T_m of 45 °C and self-aggregates with a temperature midpoint T_a of 52 °C.

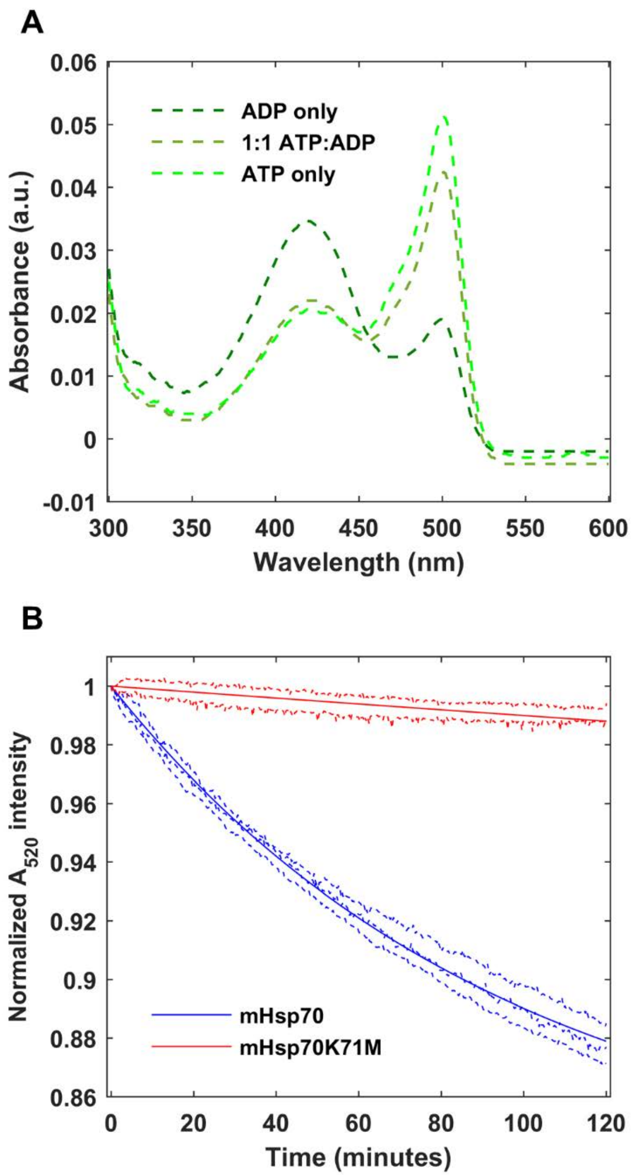

Figure B: *In vitro* measurement of mHsp70 and mHsp70K71M ATPase activity using PercevalHR. (A) Absorbance spectra of PercevalHR with 2 mM ADP (dark green), 1 mM ATP + 1 mM ADP (green) and 2 mM ATP (light green). In ATP PercevalHR shows an absorbance peak at 500 nm. As the ATP is replaced by ADP the peak at 500 nm decreases giving rise to a new absorbance peak at 420 nm. (B) ATPase activity of mHsp70 (blue) and mHsp70K71M (red) monitored by PercevalHR emission intensity at 520 nm for 500 nm excitation. The rates were fit using a 1st order reaction rate kinetics and the rate, k, = 6.31 X 10^{-1} sec^{-1} for mHsp70 and 3.69 X 10^{-2} sec^{-1} mHsp70K71M.

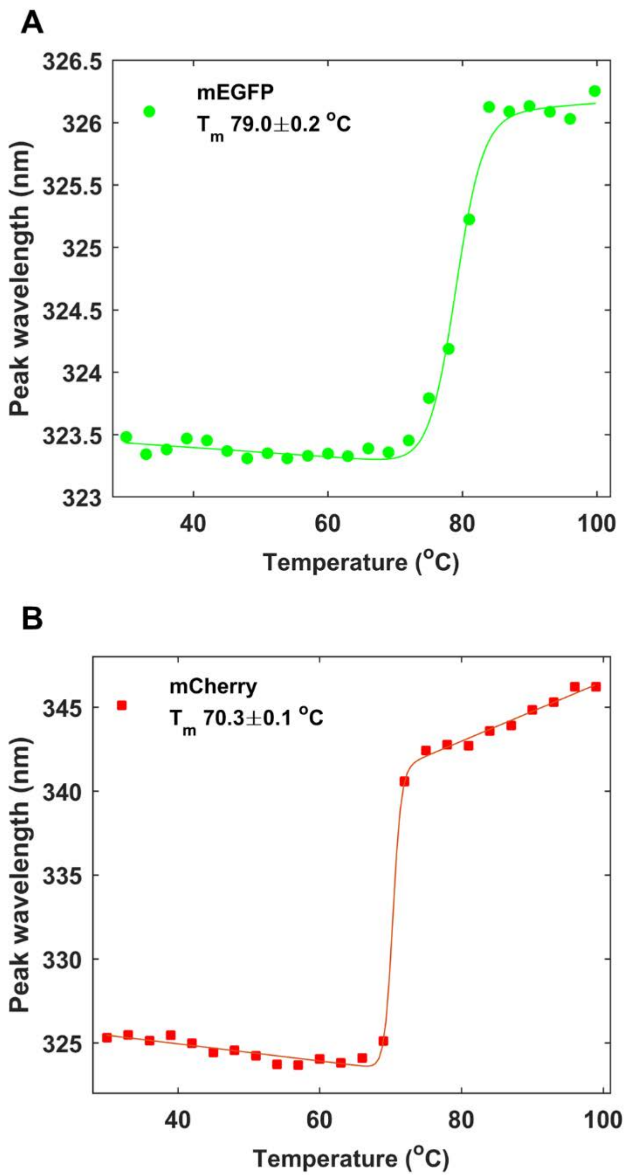

Figure C: ***In vitro*** **thermal unfolding of mEGFP and mCherry monitored by tryptophan fluorescence peak shift.** Errors shown reflect the 1 standard deviation precision of the fit. Systematic errors are not accounted for. Filled markers show experimental data and solid lines are fits to experimental data. (A) Thermal denaturation of mEGFP fit to a T_m of 79 °C. (B) Thermal denaturation of mCherry fit to a T_m of 70.3 °C.

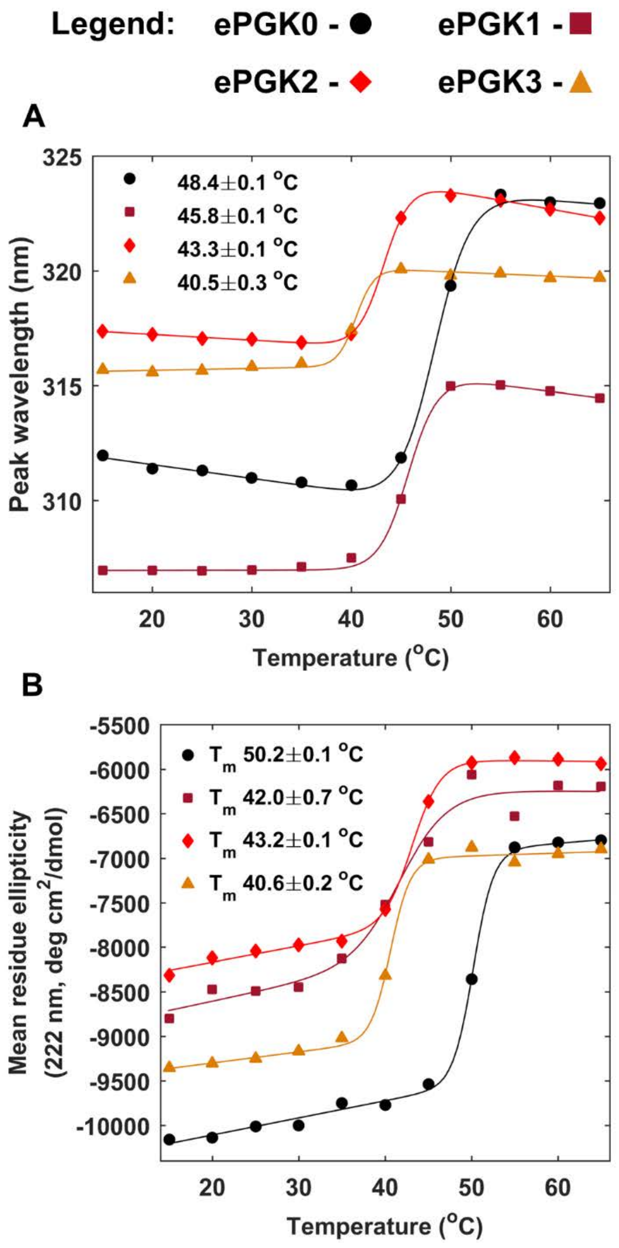

Figure D: ***In vitro* characterization of ePGKs.** Filled markers show experimental data and solid lines are fits to experimental data. Errors shown reflect the 1 standard deviation precision of the fit. Systematic errors are not accounted for. (A) Unfolding of ePGK0 through ePGK3 monitored by tryptophan fluorescence peak shift vs. temperature. The T_m fit to 48.4 °C, 45.8 °C, 43.3 °C and 40.5 °C for ePGK1 (dark red), ePGK2 (red) and ePGK3 (orange), respectively. (B) *In vitro* thermal unfolding of ePGK(0-3) monitored by circular dichroism. Thermal unfolding was monitored by change in mean residue ellipticity for ePGK0-3 at 222 nm. All unfolding curves were fit using a two-state model. The T_m fit to 50.2 °C, 42.0 °C, 43.2 °C and 40.6 °C for ePGK0 (black), ePGK1 (dark red), ePGK2 (red) and ePGK3 (orange), respectively.

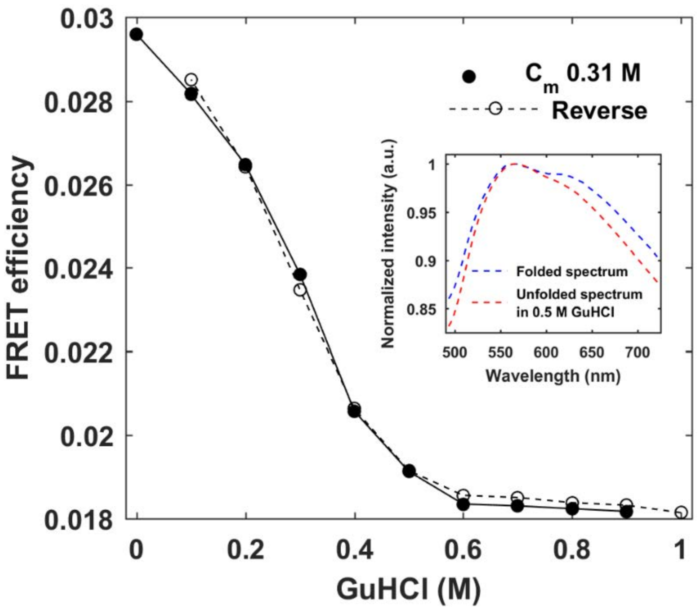

Figure E: *In vitro* FRET-PGK1 GuHCl titration. Isothermal denaturation of FRET-PGK1 with GuHCl. Markers and dashed lines show experimental data and solid lines show fits to experimental data. GuHCl titration of FRET-PGK1 (solid circles) fit to a C_m of 0.31 M. The unfolding of FRET-PGK1 in GuHCl is reversible to a large extent (open circles). Inset shows spectrum of folded FRET-PGK1 (blue) and unfolded FRET-PGK1 in 0.5 M GuHCl (red) recorded using the RSM 1000 stopped flow.

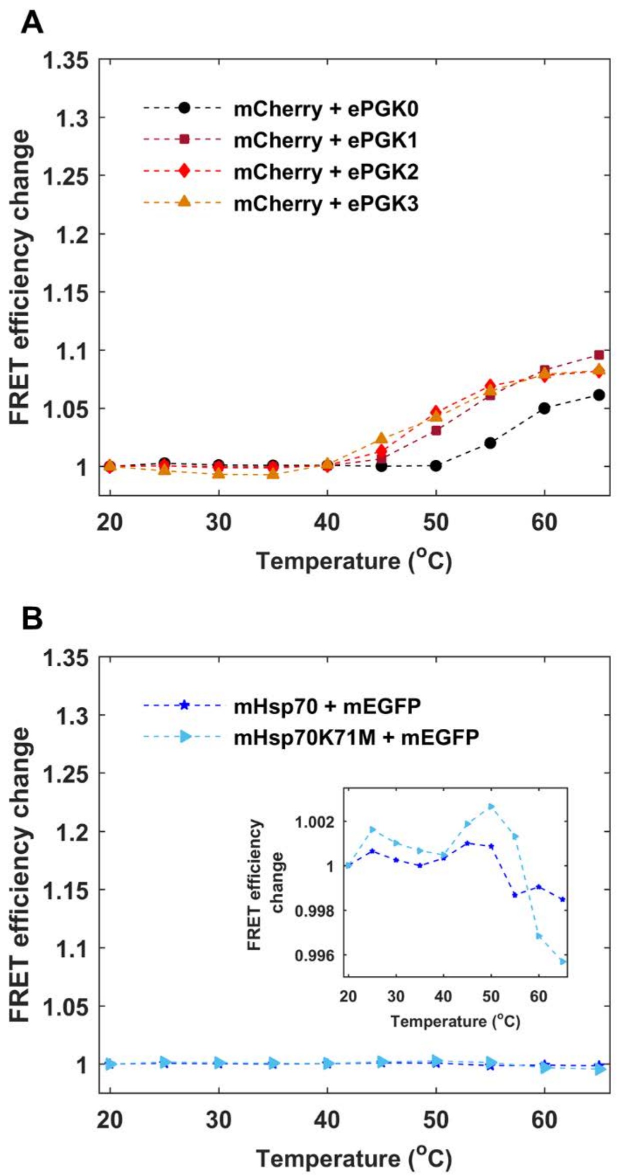

Figure F: ***In vitro*** **fluorometer controls with 5:1 mCherry:ePGKs and mHsp70:mEGFP with ATP.** Filled markers and dashed lines show experimental data. Change in FRET efficiency *vs.* temperature for (A) mCherry binding to ePGKs(0-3). A small change in FRET efficiency, 1%, was observed due to non-specific interaction between mCherry and unfolded ePGKs (B) mHsp70 binding to mEGFP. Inset shows the enlarged y-axis for mHsp70/mHsp70K71M non-specific interaction with mEGFP. The change in FRET efficiency for non-specific interaction of either unfolded mHsp70 or unfolded mHsp70K71M with mEGFP is <0.5%.

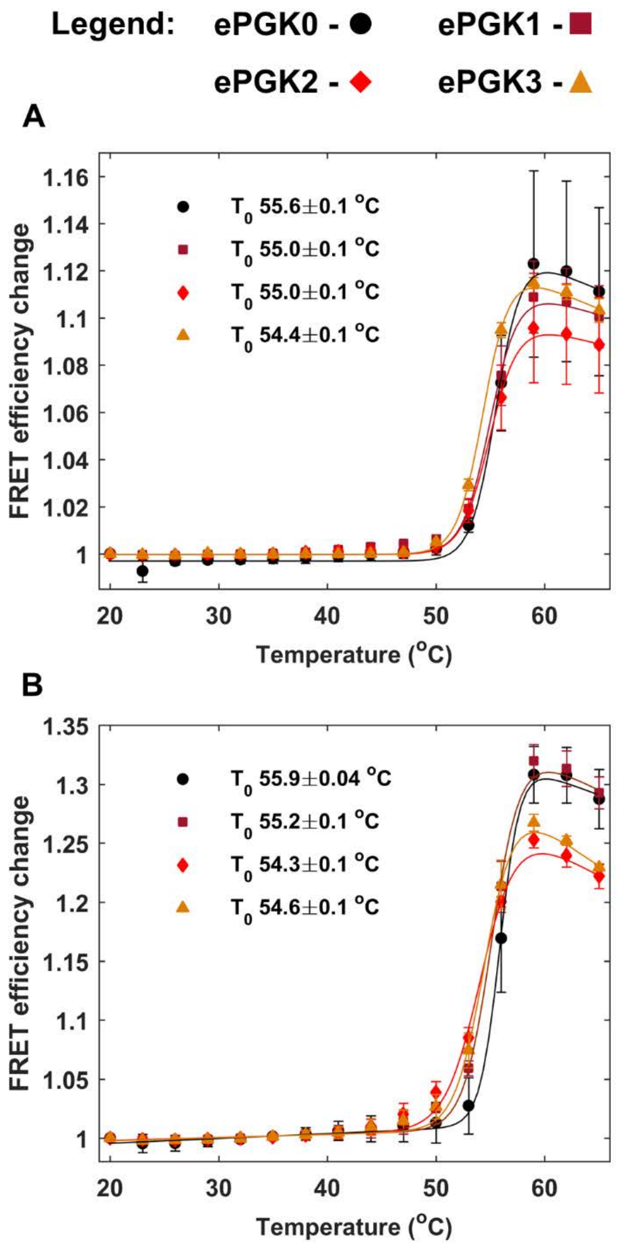

Figure G: *In vitro* binding of 1:5 and 1:1 mHsp70:ePGKs with ATP. Filled markers show experimental data and solid lines are fits to experimental data. Error bars are standard error from mean for three and two measurement repeats for panel A and B, respectively. Fit errors reflect the 1 standard deviation precision of the fit. (A) Thermal unfolding of 1:5 mHsp70:ePGKs with ATP. The binding curves for ePGK0- (black), ePGK1- (dark red), ePGK2- (red) and ePGK3-mHsp70 (orange) were fit using a two-state model and yielded a binding T_0 of 55.6 °C, 55.0 °C, 55.0 °C and 54.4 °C, respectively. (B) Thermal unfolding of 1:1 mHsp70:ePGKs with ATP. The binding curves for ePGK0- (black), ePGK1- (dark red), ePGK2- (red) and ePGK3-mHsp70 (orange) were fit using a two-state model and yielded a binding T_0 of 55.9 °C, 55.2 °C, 54.3 °C and 54.6 °C, respectively.

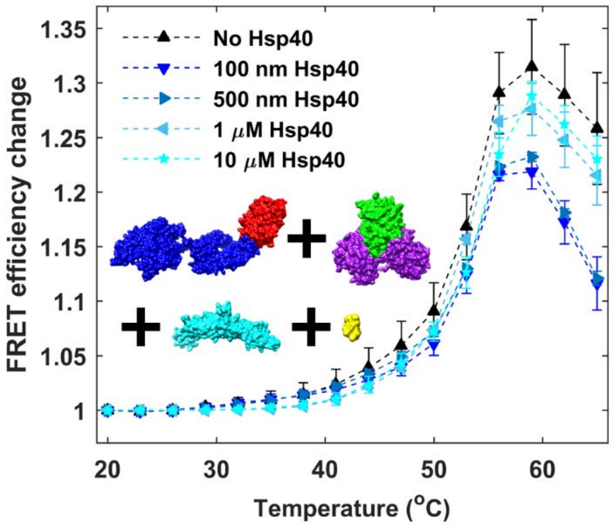

Figure H: ***In vitro*** **binding of 5:1 mHsp70 and ePGK3 with ATP and Hsp40.** Filled markers and dashed lines show experimental data. Error bars are standard error from mean for two measurement repeats. mHsp70-ePGK3 binding was conducted at multiple Hsp40 concentrations. Binding curves at all concentrations show similar change in FRET efficiency *vs.* temperature and no significant improvement in binding could be observed at any of the concentrations tested.

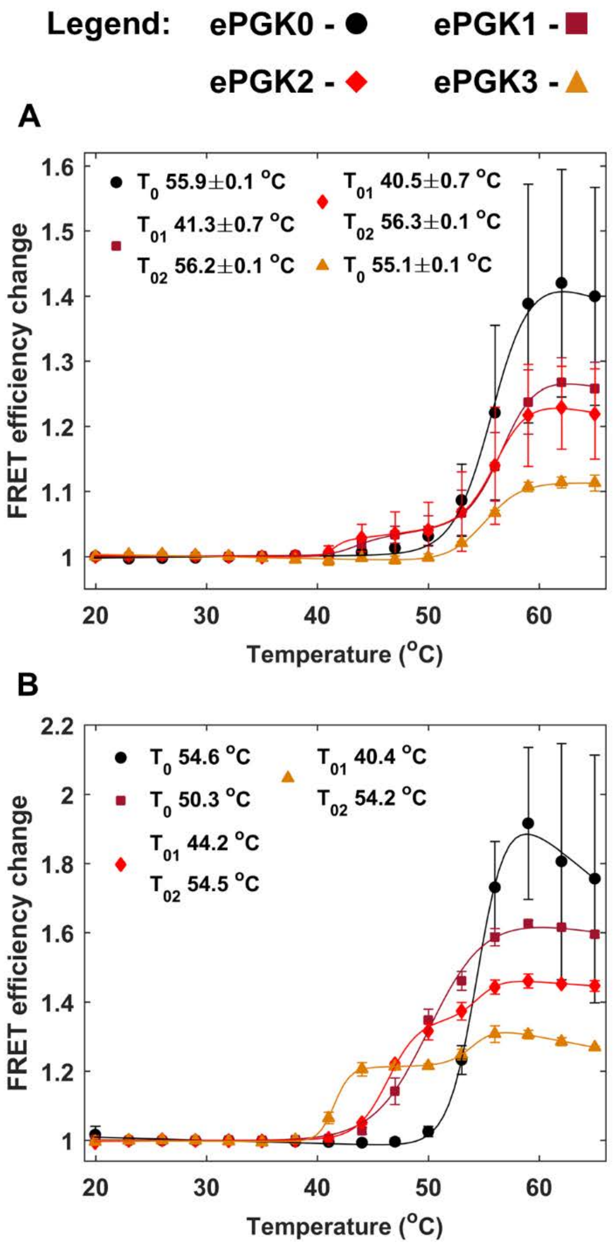

Figure I: *In vitro* binding of 1:5 and 5:1 mHsp70 and ePGKs with ATP, Hsp40 and Ficoll70. Filled markers show experimental data and solid lines are fits to experimental data. Error bars are standard error from mean for two measurement repeats. Fit errors reflect the 1 standard deviation precision of the fit. Binding of (A) 1:5 and (B) 5:1 ePGK0 (black), ePGK1 (dark red), ePGK2 (red) and ePGK3 (orange) in buffer with Ficoll70, ATP and Hsp40 with mHsp70.

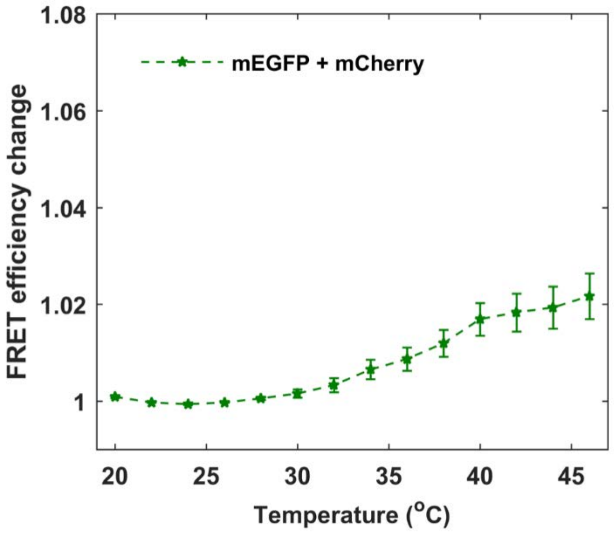

Figure J: In-cell binding of mEGFP-mCherry. The average in-cell binding FRET efficiency change for mEGFP and mCherry is only 2%, similar to the change seen for the mHsp70K71M mutant in Fig 7B, and 4x smaller than the change seen for mHsp70K71 with ePGK in Fig 7A.

Supplementary tables

Table A: Full protein sequences of proteins used. All sequences listed are wild type proteins.

**Protein (Organism)**	**Sequence**
Phosphoglycerate kinase *(Saccharomyces cerevisiae)*	MSLSSKLSVQDLDLKDKRVFIRVDFNVPLDGKKITSNQRIVAALPTIKYVLE<br>HHPRYVVLASHLGRPNGERNEKYSLAPVAKELQSLLGKDVTFLNDCVGP<br>EVEAAVKASAPGSVILLENLRYHIEEEGSRKVDGQKVKASKEDVQKFRHE<br>LSSLADVYINDAFGTAHRAHSSMVGFDLPQRAAGFLLEKELKYFGKALEN<br>PTRPFLAILGGAKVADKIQLIDNLLDKVDSIIIGGGMAFTFKKVLENTEIGDSI<br>FDKAGAEIVPKLMEKAKAKGVEVVLPVDFIIADAFSADANTKTVTDKEGIPA<br>GWQGLDNGPESRKLFAATVAKAKTIVWNGPPGVFEFEKFAAGTKALLDE<br>VVKSSAAGNTVIIGGGDTATVAKKYGVTDKISHVSTGGGASLELLEGKELP<br>GVAFLSEKK
Hsp70 *(Homo sapiens)*	MAKAAAIGIDLGTTYSCVGVFQHGKVEIIANDQGNRTTPSYVAFTDTERLI<br>GDAAKNQVALNPQNTVFDAKRLIGRKFGDPVVQSDMKHWPFQVINDGD<br>KPKVQVSYKGETKAFYPEEISSMVLTKMKEIAEAYLGYPVTNAVITVPAYF<br>NDSQRQATKDAGVIAGLNVLRIINEPTAAAIAYGLDRTGKGERNVLIFDLG<br>GGTFDVSILTIDDGIFEVKATAGDTHLGGEDFDNRLVNHFVEEFKRKHKK<br>DISQNKRAVRRLRTACERAKRTLSSSTQASLEIDSLFEGIDFYTSITRARFE<br>ELCSDLFRSTLEPVEKALRDAKLDKAQIHDLVLVGGSTRIPKVQKLLQDFF<br>NGRDLNKSINPDEAVAYGAAVQAAILMGDKSENVQDLLLLDVAPLSLGLE<br>TAGGVMTALIKRNSTIPTKQTQIFTTYSDNQPGVLIQVYEGERAMTKDNNL<br>LGRFELSGIPPAPRGVPQIEVTFDIDANGILNVTATDKSTGKANKITITNDK<br>GRLSKEEIERMVQEAEKYKAEDEVQRERVSAKNALESYAFNMKSAVEDE<br>GLKGKISEADKKKVLDKCQEVISWLDANTLAEKDEFEHKRKELEQVCNPII<br>SGLYQGAGGPGPGGFGAQGPKGGSGSGPTIEEVD
Hdj1 (Hsp40) *(Homo sapiens)*	MGKDYYQTLGLARGASDEEIKRAYRRQALRYHPDKNKEPGAEEKFKEIA<br>EAYDVLSDPRKREIFDRYGEEGLKGSGPSGGSGGGANGTSFSYTFHGD<br>PHAMFAEFFGGRNPFDTFFGQRNGEEGMDIDDPFSGFPMGMGGFTNVN<br>FGRSRSAQEPARKKQDPPVTHDLRVSLEEIYSGCTKKMKISHKRLNPDG<br>KSIRNEDKILTIEVKKGWKEGTKITFPKEGDQTSNNIPADIVFVLKDKPHNIF<br>KRDGSDVIYPARISLREALCGCTVNVPTLDGRTIPVVFKDVIRPGMRRKVP<br>GEGLPLPKTPEKRGDLIIEFEVIFPERIPQTSRTVLEQVLPI
mEGFP	MVSKGEELFTGVVPILVELDGDVNGHKFSVSGEGEGDATYGKLTLKFICT<br>TGKLPVPWPTLVTTLTYGVQCFSRYPDHMKQHDFFKSAMPEGYVQERTI<br>FFKDDGNYKTRAEVKFEGDTLVNRIELKGIDFKEDGNILGHKLEYNYNSH<br>NVYIMADKQKNGIKVNFKIRHNIEDGSVQLADHYQQNTPIGDGPVLLPDN<br>HYLSTQSKLSKDPNEKRDHMVLLEFVTAAGITLGMDELYK
mCherry	MVSKGEEDNMAIIKEFMRFKVHMEGSVNGHEFEIEGEGEGRPYEGTQTA<br>KLKVTKGGPLPFAWDILSPQFMYGSKAYVKHPADIPDYLKLSFPEGFKWE<br>RVMNFEDGGVVTVTQDSSLQDGEFIYKVKLRGTNFPSDGPVMQKKTMG<br>WEASSERMYPEDGALKGEIKQRLKLKDGGHYDAEVKTTYKAKKPVQLPG<br>AYNVNIKLDITSHNEDYTIVEQYERAEGRHSTGGMDELYK

Table B: Transition temperature mid-points for Hsp70 and mHsp70 calculated from two-state sigmoidal fits to the experimental data. Errors shown reflect the 1 standard deviation precision of the fit. Systematic errors are not accounted for.

Protein	Nucleotide	Nucleotide concentration (mM)	Transition temperature (°C)		
			CD	Trp SVD	FRET
Hsp40	NA	0	56.2 ± 0.3	NA	NA
Hsp70	NA	0	45.8 ± 0.9	47.3 ± 0.2	NA
Hsp70	ATP	20	NA	52.4 ± 0.1	NA
Hsp70-mCherry	NA	0	45.8 ± 0.5	44.4 ± 0.3	45.5 ± 0.1
Hsp70-mCherry	ATP	20	NA	53.5 ± 0.2	55.2 ± 0.1
Hsp70-mCherry	ADP	20	NA	53.5 ± 0.4	NA

Table C: Melting temperatures and point mutations for PGKs tested. T_m was estimated from two-state sigmoidal fits to the experimental data. Errors shown reflect the 1 standard deviation precision of the fit. Systematic errors are not accounted for.

Protein	Mutations (Y122W+)	Melting T_m (°C)		In-cell binding T_0 (°C)
		CD	Trp peak shift	FRET
mEGFP-PGK0	P111T	50.4 ± 0.3	48.4 ± 0.1	>46
mEGFP-PGK1	W308F, W333F	43.2 ± 0.5	45.8 ± 0.3	>41.0 ± 0.4
mEGFP-PGK2	P111T, P204H	43.3 ± 0.4	43.3 ± 0.1	39.1 ± 0.1
mEGFP-PGK3	W308F, P204H	41.0 ± 0.4	40.5 ± 0.3	37.1 ± 0.2

Table D: Binding T_0 for *in vitro* binding of mHsp70-ePGK. T_0 was estimated from two- or three-state sigmoidal fits to the experimental data. Errors shown reflect the 1 standard deviation precision of the fit. Systematic errors are not accounted for.

Protein	ATP only		ATP + Hsp40 + Ficoll		ATP + Hsp40 + Ficoll + cell lysate	
	T_{01} (°C)	T_{02} (°C)	T_{01} (°C)	T_{02} (°C)	T_{01} (°C)	T_{02} (°C)
mEGFP-PGK0	-	55.2 ± 0.2	-	55.2 ± 0.2	-	55.0 ± 0.5
mEGFP-PGK1	-	54.2 ± 0.4	-	51.6 ± 0.1	-	51.7 ± 0.3
mEGFP-PGK2	-	53.0 ± 0.5	43.3 ± 0.9	53.3 ± 0.1	-	50.6 ± 0.7
mEGFP-PGK3	40.0 ± 0.8	54.1 ± 0.4	40.0 ± 1.0	52.0 ± 0.1	41.0 ± 0.4	55.4 ± 0.6
