# Supplementary material for: Heat shock-induced chaperoning by Hsp70 is enabled in-cell
Source: PLoS One. 2019 Sep 26;14(9):e0222990. doi: 10.1371/journal.pone.0222990 (PMC6762143; doi:10.1371/journal.pone.0222990)
Supplement: Attachment — Submitted filename: Reviewer_response.pdf [file pone.0222990.s002.pdf]

Dear José,

We have completed the revision for PONE-D-19-15421. It took a little extra time because we had to make an additional measurement requested (very reasonably) by reviewer #1. (A co-author was added for these measurements.) Below you'll find a summary of the reviewer comments and the changes made in response. We hope that with these changes, the manuscript will be suitable for publication in PlOS1.

Best,

Martin

Financial Disclosure:
This work was supported by NSF grant MCB 1803786.

Reviewer #1:

The authors investigate how the molecular chaperone Hsp70 binds a client protein, PGK, during heat shock. Their FRET studies show that while binding is non-specific in the test tube, there is some specific binding in cells that likely occurs as hydrophobic regions in PGK are exposed during heating. This manuscript provides an important glimpse into how the Hsp70 class of molecular chaperones protect proteins from heat shock in cells and should be published after some revisions.

Equations 5 accounts for the unfolded state of the client protein, the chaperone bound client protein and the aggregates of the client-chaperone complex. But, especially under heat shock one might expect the unbound client to aggregate even when not chaperone bound. Is there a reason to exclude this aggregated species from the analysis?

Author response: The authors thank the reviewer for the helpful comment. When all our PGK constructs are FRET-labeled (with two labels) so as to probe protein folding, a small signature of aggregation is indeed observed around 50 °C. See figure below, where unfolding leads to a decrease of the FRET efficiency, but a small increase after unfolding is visible at ~52 °C due to aggregation. We have to neglect this state for two reasons: (1) It does not have a significant signal in our experiment because our PGK is only singly-labeled, not FRET-labeled; (2) in the experiments with ATP, the binding occurs below 45 °C, before the PGK aggregation makes a significant contribution. Even though some PGK is likely self-aggregated at high temperatures after unfolding, we could not get well-defined fits by including aggregated PGK states, and hence they have not been accounted for in the simplified equation 5. Line 274-278 of the main text now explains this, as well as Figure S1 (also shown below).

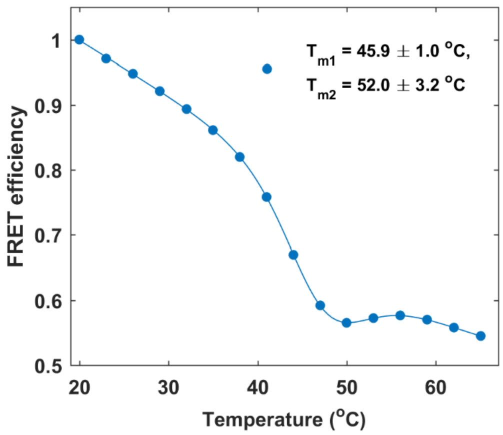

The Hsp70 and Hsp40 constructs used for these experiments are the human molecular chaperones, but the PGK construct is from yeast. What is the percent identity between human PGK and the wild-type PGK construct used in these experiments? Also, for figure 8, how were the green "hydrophobic patches" identified? Do these regions overlap with regions that might bind Hsp70 based on the Limbo server (http://limbo.switchlab.org/limbo-analysis)? The Limbo server is based on the binding propensities of the E. coli Hsp70 DnaK, nonetheless, these predictions might be useful in considering what regions of PGK might be involved in binding human HspA1A.

Author response: The authors thank the reviewer for the helpful suggestion of the limbo website. Yeast PGK and human PGK share 65% sequence identity and 76% sequence similarity (Emboss Needle alignment tool). This line was also added to the main text (lines 345-347). The figure 8 was re-plotted as Figure 9 to show the predictions made using the limbo website and it was added in the results (Line 502-504).

In Figure 2, the authors show that the Hsp70/Hsp40 system can improve PGK refolding efficiency following denaturation in guanidine hydrochloride and they use these data to conclude "Thus, PGK1 refolding from denaturant is chaperoned by Hsp70 and eukaryotic PGK is an Hsp70 substrate." However, they later show that at elevated temperature in vitro interactions between PGK and Hsp70 are non-specific. Thus, it seems possible that the more efficient refolding also arises due to a non-specific interaction. Does the K71M Hsp70 mutant have the same effect? (If this experiment was not performed it is sufficient to replace "is an Hsp70 substrate" with something like "appears to be..")

Author response: This turned out to be a very important suggestion. We added an additional experiment to figure 3 comparing recovery with wt-Hsp70 vs. the ATPase-deficient mutant Hsp70K71M. PGK is indeed a non-specific substrate for Hsp70 both by heat shock AND by denaturant. Lines 25, 38, 100-109, 367-373, 512 were changed accordingly. We also added a small section under methods detailing the fitting function lines 290-297. We also did the new stopped flow experiments with mEGFP as the donor instead of AcGFP1 as reported previously, to make all *in vitro* and in-cell data even more directly comparable. Thus Line 147-149 now says mEGFP instead of AcGFP1. Our results show that indeed PGK is a non-specific substrate even by stopped flow. Thus neither *in vitro* condition, heat shock or denaturant, yielded specific binding, but specific binding was rescued in the cell. We thinks this is an even more exciting result (and hope the reviewer agrees!), as it points to the possibility of Hsp70 having an even wider palette of substrates in-cell than *in vitro*, as we now point out in the Discussion and Conclusions.

Page 2 line 59: "ATP hydrolysis is induced by substrate binding….." This description is not quite correct. Hsp70s have a basal rate of ATP hydrolysis which is increased (stimulated) by substrate binding.

Author response: The statement was fixed according to reviewer comment.

Page 4 lines 106-107 "There is a precedent for such native state binding activity for the Hsp70 homolog DnaK, where the chaperone binds to full-length native proteins in force-unfolding experiments [25]." This reference is not correct – likely it should be reference 24.

Author response: The reference was fixed.

Page 6 Line 179: "Tryptophan was excited at 295 nm, and emission spectra were collected from 290 to 450 nm." Either the excitation wavelength or emission range is likely incorrect.

Author response: Generally, our samples with ATP are excited at 295 nm to avoid the intense ATP absorbance at 280 nm, and maximize the tryptophan over the tyrosine contribution to the fluorescence. We start collecting emission at 290 nm anyway, which overlaps with the excitation as pointed out by the reviewer. This is done so we can monitor the excitation peak for signs of aggregation: formation of

aggregates leads to scatter and a drastic change in excitation signal. If this occurs, the experiments are repeated. This clarification is added to the methods (line 180-184).

Reviewer #2:

The authors compare in-vitro and in-cell measurements of heat shock proteins binding to its substrate and find specific differences. They attribute these differences to in-cell conditions that promote a 'preemptive holdase' mechanism for the chaperone to bind and not promote unfolding. The experiments have been performed carefully and the manuscript is written well. I have the following minor comments:

1) The authors should report the fitting errors for the various parameters including Tm, T0, fast phase and slow phase time-constants.

Author response: An additional table S4 was added that shows errors for in vitro fits. Both tables S3 and S4 show all fit errors referenced in the main text in line 348 and 445.

2) How are the surface exposed sites identified in Figure 8?

Author response: Originally the authors reported any 7 aa long hydrophobic stretches in the protein. However, following the suggestion of reviewer 1, the website **http://limbo.switchlab.org/limbo-analysis** was used to predict binding sites. Limbo predicts binding sites for the bacterial Hsp70, DnaK, on a potential substrate. The overall pattern is similar, but the limbo-predicted patterns in the new Figure 9 are probably more accurate than our previous version of Figure 8.

3) It would be good to provide a cartoon that summarizes the result given that multiple proteins and conditions have been explored.

Author response: A new Figure 8 summarizes the overall results discussed in the paper.

4) The amplitudes of the unfolding curves are very different. Do they have any mechanistic origins in terms of binding or folding or aggregation?

Author response: Indeed, the maximum changes in FRET efficiency from Hsp-PGK binding vary between 1.1 and 1.3 – not counting the large changes at > 50° from Hsp-self aggregation. We believe these differences are caused by changes in crowding (buffer vs. Ficoll vs. in-cell) and binding sites (specific ATP-dependent vs. non-specific binding), but currently no structural models are available to quantify this. We discuss it now briefly in the Discussion and Conclusions section (lines 520-522).
